# Comparison of the results of open PLIF versus UBE PLIF in lumbar spinal stenosis: postoperative adjacent segment instability is lesser in UBE

**DOI:** 10.1186/s13018-023-04038-3

**Published:** 2023-07-29

**Authors:** Xiaobin Li, Jie Liu, Zhiwei Liu

**Affiliations:** 1 The Third Affiliated Hospital of Soochow University, Changzhou, China; 2 Yangzhou University, Yangzhou, China

**Keywords:** Minimally invasive surgery, Lumbar spinal stenosis, Instability, Adjacent, Endoscopic, Fusion

## Abstract

**Objective:**

To compare the difference in efficacy between open PLIF and UBE for lumbar spinal stenosis and the effect on postoperative adjacent segment instability.

**Method:**

The clinical data of 37 patients with PLIF and 32 patients with UBE for lumbar spinal stenosis were retrospectively analyzed to compare the differences in perioperative conditions and short- and medium-term outcomes.

**Results:**

All 69 patients completed the surgery successfully. The operating time, number of intraoperative fluoroscopies and hospital days were higher in the UBE group than in the open PLIF group. Intraoperative bleeding and postoperative drainage were lower than in the open PLIF group (*P* < 0.05). The visual analogue scale (VAS) of low back pain was lower in the UBE group than in the open PLIF group at 1 month and 3 months postoperatively (*P* < 0.05), and there were no statistically significant VAS scores for low back pain in the two groups at 1 day and 6 months postoperatively (*P* > 0.05). Leg pain VAS scores were lower in the UBE group than in the open PLIF group at 1 month, 3 months and 6 months postoperatively (*P* < 0.05), and leg pain VAS scores were not statistically significant in both groups at 1 day postoperatively (*P* > 0.05). The ODI index was lower in the UBE group than in the open PLIF group at 1 day and 1 month postoperatively (*P* < 0.05) and was not statistically significant in the two groups at 3 months and 6 months postoperatively (*P* > 0.05). There was no statistically significant difference between the two groups in postoperative interbody height, sagittal diameter of the spinal canal, efficacy of modified MacNab and interbody fusion (*P* > 0.05). The open PLIF group was more prone to postoperative adjacent vertebral instability than the UBE group, and the difference was statistically significant (*P* < 0.05).

**Conclusion:**

With appropriate indications, the open PLIF group and the UBE group had similar short- and medium-term clinical outcomes for the treatment of lumbar spinal stenosis, but patients in the UBE group had better symptomatic improvement than the open PLIF group at 3 months postoperatively, and the effect on postoperative adjacent vertebral instability was smaller in the endoscopic group than in the open PLIF group.

## Introduction

In recent years, the incidence of lumbar spinal stenosis (LSS) has increased year by year and has become one of the most common diseases in the world, with approximately 103 million patients diagnosed each year, and the disease is more common in developing countries, and the incidence in China is 3.5 times higher than that in the USA [1]. As the modern population changes the way they work,progressively younger age of onset. Lumbar spinal stenosis can lead to back and leg pain, seriously affecting daily life such as walking. Eventually, they can become disabled and have a significant socioeconomic impact. Conservative treatment is difficult to improve the symptoms of some patients. Currently, vertebral fusion surgery is considered to be an effective treatment. It can significantly improve patients’ symptoms such as back and leg pain and improve their quality of life [2]. The traditional open surgical procedure commonly used is the posterior lumbar column fusion. The posterior column structures include the interspinous ligaments, the small articular synapses and the joint capsule. Studies have demonstrated that they are extremely important in maintaining spinal stability and that their functional integrity determines postoperative outcomes. However, open surgery tends to cause extensive damage to the surrounding skin and muscle tissue and destabilize the posterior column structure. This leads to further disk degeneration, facial neuropathy, perioperative facial nerve injury, screw encroachment on the proximal fracture joint, facial nerve fracture, stress transition and bone destruction [3]. Therefore, reducing damage to paravertebral muscles and posterior column structures is a key factor in long-term outcomes [4]. Recent studies have shown that minimally invasive surgery is the best approach for the treatment of LSS [5]. And with advances in surgical instrumentation and endoscopic techniques, minimally invasive spine surgery has rapidly evolved from small incisions to tubular or percutaneous endoscopy. Therefore, in addition to maintaining the integrity of the posterior column, minimally invasive surgery has the advantages of smaller wounds, less local pain, less blood loss and shorter hospital stays compared to traditional open surgery. Many clinical studies and meta-analyses in recent years have confirmed the effectiveness and safety of minimally invasive lumbar interbody fusion [6, 7]. It has been found that after lumbar fusion, instability can occur in adjacent vertebrae due to changes in stress relationships [8]. Vertebral instability is due to pathological changes caused by the inability of the vertebral body position to maintain normal positional relationships under normal loading [9]. There are no specific signs or symptoms in the early stages, which can only be detected by radiological data on postoperative review; among them, lumbar flexion and extension radiography is the most widely used [10]. Therefore, the purpose of this paper is to compare the perioperative situation, the difference in short- and medium-term outcomes, and the magnitude of the effect on postoperative adjacent vertebral instability between open lumbar interbody fusion and endoscopic lumbar interbody fusion, in order to provide a reference for selecting the appropriate treatment for the patient.

## Materials and methods

### Research subjects

In this study, we retrospectively analyzed the data of patients with lumbar spinal stenosis treated at the Department of Spine Surgery of the Third Affiliated Hospital of Soochow University from June 2021 to August 2022. This study was approved by the Ethics Committee of Changzhou First People’s Hospital. Patient selection is based on the following criteria.

*Inclusion criteria* are: (1) clear preoperative diagnosis of lumbar spinal stenosis with lesion segments L3-L4 or L4-L5; (2) no significant relief after more than 6 months of preoperative conservative treatment; and (3) patient with disk prolapse or LRS or central stenosis or foraminal stenosis or instability.

**Fig. 1:**
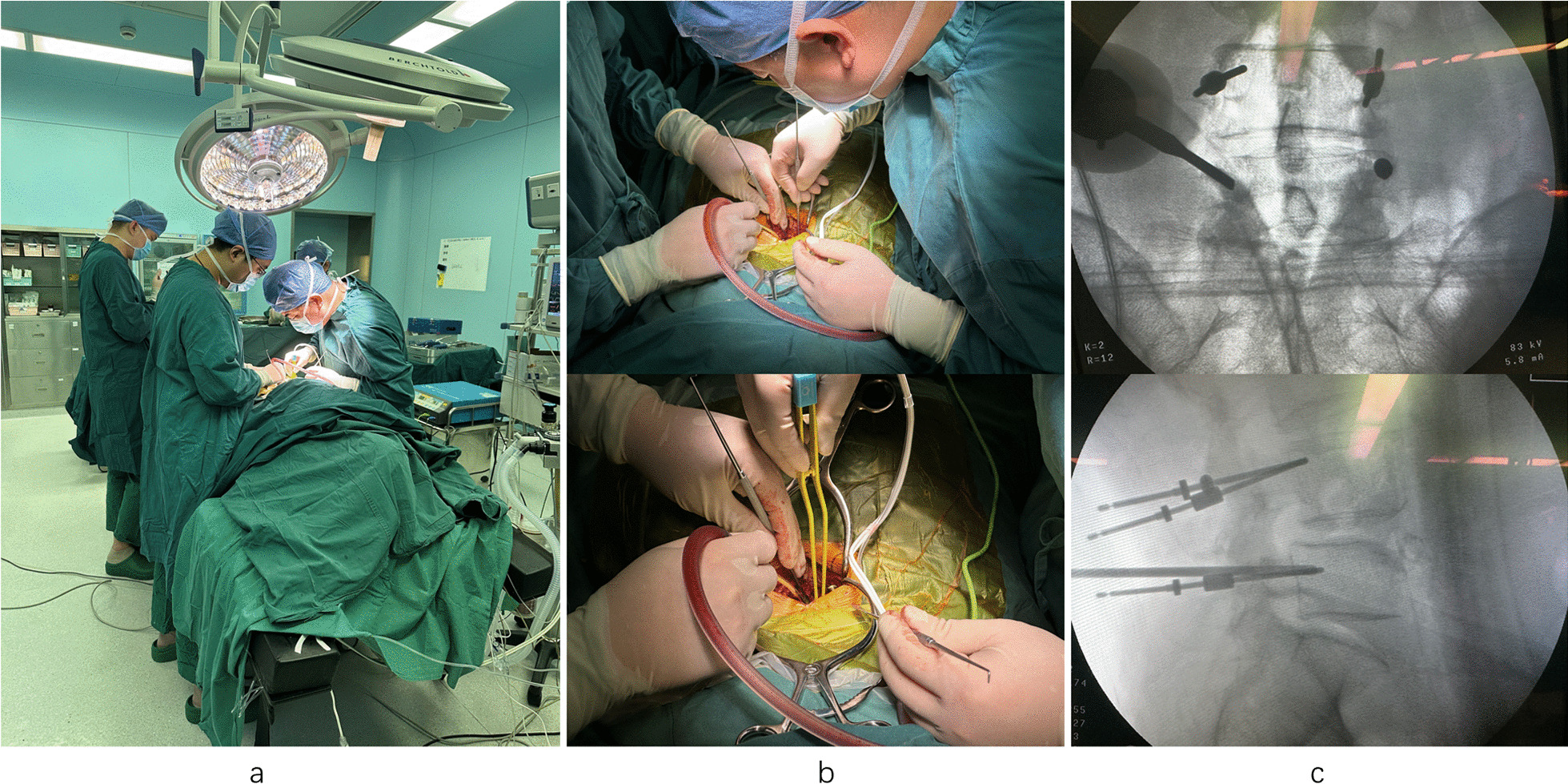
PLIF (intraoperative). **a** The doctors are operating. **b** The doctors are reducing the pressure. c: The screw position was good by intraoperative fluoroscopy

*Exclusion criteria* are: (1) previous lumbar spine surgery; (2) combined with other segmental disk herniation with instability, lesion instability is not clear; (3) combined with tuberculosis, infection, tumor; (4) combined with cauda equina syndrome symptoms; and (5) receiving anticoagulation or antiplatelet drugs within 6 months (Fig. 1).

### Preoperative preparation

Patients underwent preoperative routine blood and biochemical examinations, lumbar spine X-ray, CT and MR imaging. The patient was placed in the prone position, under general anesthesia, with close monitoring of vital signs and attention to the protection of the airway, and routine disinfection and towel laying.

### Surgical method

#### Open PLIF

1. After satisfactory anesthesia, take the prone position and routinely disinfect the sheet.
2. First of all, determine a good surgical plane, take a posterior median lumbar incision, about 10 cm long, cut the skin and deep fascia, cut the paravertebral muscles on both sides of the spinous process, expose the lumbar vertebral plate, upper and lower articular processes and transverse processes, the intersection of the outer edge of the upper articular process and the midline of the transverse process as the opening point, the opener is played into the vertebral body through the arch, the spinal navigation shows a good length position, and the arch nail is driven into the arch.
3. Removal of the spines of the cone, exposure of the intervertebral space and upper and lower vertebral plates, resection of the inferior 2/3 of the superior vertebral plate and the superior 1/3 of the inferior vertebral plate, and the intra-articular 1/3 of the superior synovial process. Expose the ligamentum flavum, peel off the ligamentum flavum, and then, remove it completely. The dura mater and the nerve roots on both sides are exposed and retracted centrally, the free nucleus pulposus is removed, the intervertebral disk is excised, and the endplate cartilage is scraped. A sufficient amount of autogenous bone is implanted in the intervertebral space, followed by the implantation of a fusion device. The distal nerve root canal was explored without stenosis, nerve root relaxation was observed, the wound was irrigated, and strict hemostasis was achieved. One connecting rod is placed at the end of the left pedicle nail and the other at the end of the right pedicle nail, and then, the end cap is tightened.
4. Good fixation was checked and the internal fixation system was well positioned by C-arm fluoroscopy. After rinsing the wound and strict hemostasis, the bone graft bed was prepared with bone chisels on both sides of the transverse process of the cone, and then, a mixture of autologous and allogeneic bone was implanted, and two silicone balls were built into the wound to drain it and finally sutured layer by layer.
5. The procedure went well, anesthesia was good, and the dressing was wrapped and returned to the ward.

#### UBE

1. After successful anesthesia, the patient was placed in the prone position and the towel was routinely disinfected.
2. Firstly, the cone was marked on the bilateral arch projection, and the arch puncture was performed with a puncture needle at 1 cm from the bilateral projection of the vertebral body, and the C-arm guidance showed that the arch puncture position was satisfactory, and the position was confirmed by fluoroscopy again after placing six guidewires.
3. Then determine the plane of surgery. A 1.2 cm oblique incision is made in the left side of the vertebral body above the left projection of the pedicle. The endoscope is placed after percutaneous transdermal peeling of the subcutaneous soft tissue. Endoscopically assisted resection of the articular eminence and part of the lamina of this intervertebral space.
4. Exploration of the nerve root and release of the nerve root canal, which was released and seen to have no significant compression. Pull the dura medially to the radiofrequency electrode to stop the bleeding, and then, swap channels, using the staple incision as the viewing window and the caudal incision as the operating window. Using the UBE technique, the intervertebral space is treated with alternating open reamers and spatulas under endoscopic surveillance. The proximal lamina is treated up to the inferior stop of the proximal lamina of the ligamentum flavum. Treatment of the ligamentum flavum at the superior edge of the vertebral plate of the inferior vertebral body. Removal of the entire ipsilateral ligamentum flavum. The bone is then implanted into the intervertebral space and a BMP is applied, and finally, the fusion is placed. The fusion was seen on fluoroscopy to be in satisfactory position, and the surgical area was flushed with plenty of saline at the end of the procedure.
5. The procedure went well, anesthesia was good, and the dressing was wrapped and returned to the ward.

### Postoperative treatment

1. Postoperative treatment such as pain relief, dehydration and swelling reduction, and nerve nutrition were given; routine blood tests and blood biochemistry were repeated on the first postoperative day.
2. Patients were instructed to do axial turning, functional exercises of both lower limbs and ankle pump during bed rest to prevent bed-related complications such as lower limb venous thrombosis.
3. Drainage flow should be recorded daily, and the drainage tube should be removed only when it is less than 50 mL/d.
4. After the drainage tube is removed, the lumbar spine X-ray frontal and lateral radiographs are reviewed on the same day; the lumbar brace can be worn to move on the ground appropriately.
5. The patient was instructed to wear a lumbar brace for at least 3 months and to review the lumbar spine X-ray frontal and lateral radiographs 1 to 6 months after surgery and lumbar spine CT if necessary.

### Evaluation indexes

**Fig. 2:**
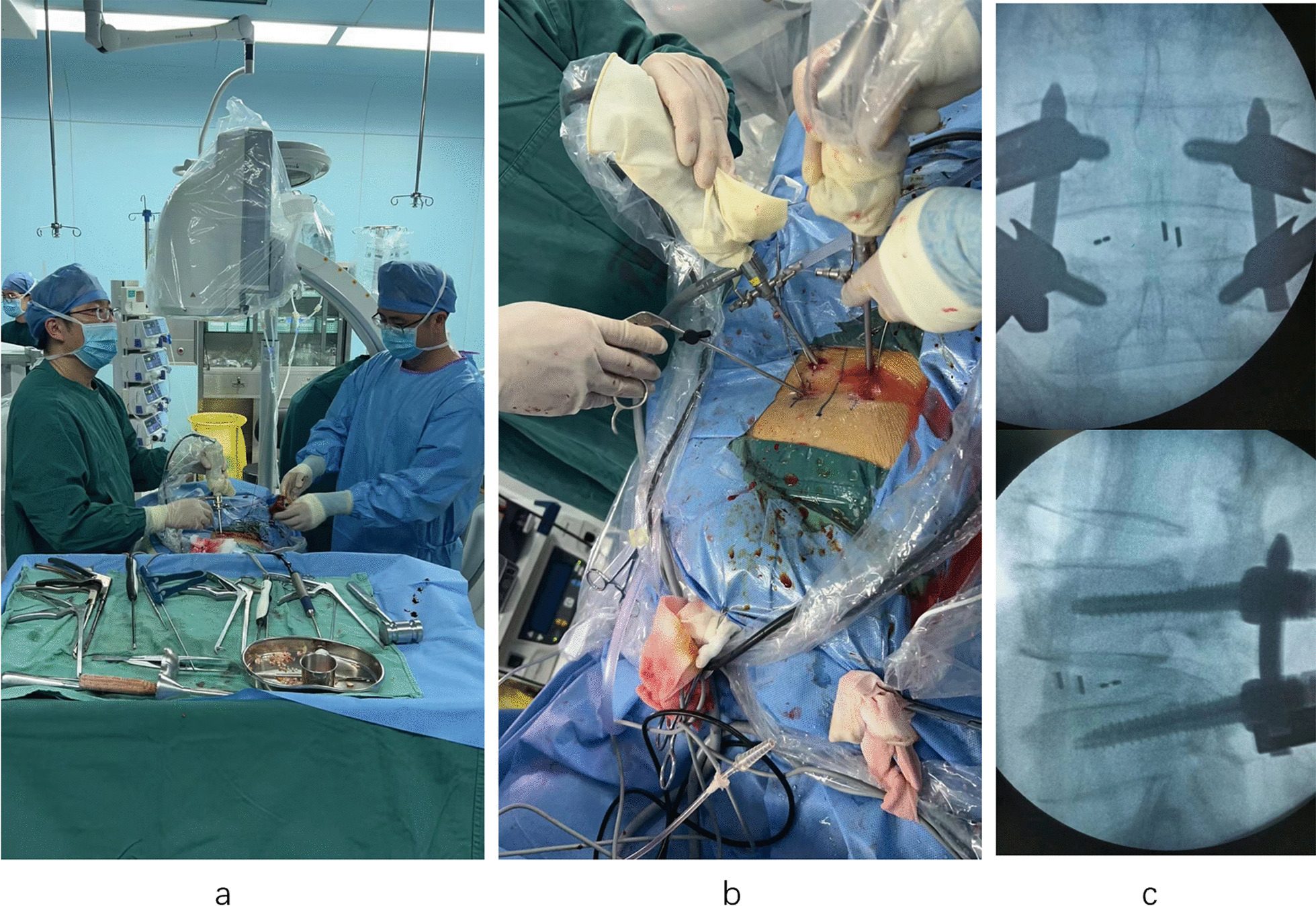
UBE (intraoperative). **a** The doctors are operating. **b** Decompression was performed simultaneously on both sides. **c** The screw position was good by intraoperative fluoroscopy

One doctor recorded the data of two groups of patients. This includes operative time, number of intraoperative fluoroscopies, intraoperative blood loss, postoperative drainage, hospital days and complication rate. The VAS scores for low back pain and leg pain were recorded by the same physician before, 1 day after, and 1, 3 and 6 months after surgery. The ODI indices were recorded by the same physician preoperatively, 1 day postoperatively, and 1, 3 and 6 months postoperatively. The intervertebral space height and sagittal diameter of the spinal canal were measured in both groups of patients before and 6 months after surgery according to lumbar spine imaging data (including X-ray or CT or MRI). The efficacy of the modified MacNab was recorded at 6 months postoperatively. Intervertebral fusion was assessed using the Brantigan–Steffee criteria. Each patient was observed for wound healing grade prior to discharge (Fig. 2).

Radiographic manifestations of vertebral instability are: (1) displacement > 3–4 mm and angle formation > 9–10°; (2) changes in the vertebral tubercle in the plane of slippage: sclerosis of the articular surface, narrowing of the vertebral tubercle and hypertrophy or asymmetry of the articular processes; (3) changes in the adjacent vertebral body and vertebral space: osteophytes at the edge of the vertebral body, hypertrophy and hyperplasia of the joint, and narrowing of the vertebral space; and (4) dynamic radiography: the relative displacement distance between vertebral bodies is greater than or equal to 3.0 mm, the maximum is 20 mm.

### Statistical methods

Data analysis was performed using SPSS 25.0 software comparison between groups and multiple comparisons. The counts were tested by Chi-square test. Rank-sum test was used for rank information. Independent samples *t*-test was used for measurement data. *P* < 0.05 was considered a statistically significant difference.

## Results

### Perioperative clinical information

A total of 69 patients eventually met the enrollment criteria. Patients were divided into two groups, depending on the type of surgical placement. The open PLIF group consisted of 37 patients who underwent open lumbar interbody fusion. The UBE group consisted of 32 patients who underwent endoscopic lumbar interbody fusion. The differences in age (*t*-test) and gender (Chi-square test) between the two groups were not statistically significant (*P* > 0.05). See Table 1.

**Table 1:** General information of patients in both groups

Group	Number of cases	Gender (M/F)	Age ($\overline{x} \pm s$, year)
Open group	37	22/15	63.46 ± 10.96
Endoscopic group	32	15/17	62.47 ± 9.57
*x*^2^/*t*		1.092	0.397
*P*		0.296	0.693

Both groups completed the surgery successfully. The incisions were all grade A healed. The operating time, number of intraoperative fluoroscopies and number of hospital days were significantly greater in the UBE group than in the open PLIF group (*P* < 0.05). Intraoperative bleeding and postoperative drainage in the UBE group were significantly smaller than those in the open PLIF group (*P* < 0.05). See Table 2.

**Table 2:** Comparison of perioperative indicators

Group	Surgery time (h)	Intraoperative bleeding (ml)	Number of intraoperative fluoroscopies	Postoperative drainage (ml)	Length of hospitalization (d)
Open group	2.20 ± 0.75	221.62 ± 113.37	2.05 ± 0.81	611.86 ± 265.33	15.81 ± 3.24
Endoscopic group	4.31 ± 1.41	114.06 ± 31.71	3.84 ± 0.45	85.84 ± 78.4	17.28 ± 2.61
*t*	− 7.914	5.190	− 11.059	10.805	− 2.056
*P*	0.000	0.000	0.000	0.000	0.044

### Efficacy evaluation

#### VAS score for low back pain

The difference in preoperative low back pain VAS scores between the two groups was not statistically significant (*P* > 0.05). The postoperative VAS scores of low back pain were lower in both groups compared with the preoperative scores, and the difference was statistically significant (*P* < 0.05). The VAS scores of low back pain in the UBE group were lower than those in the open group at 1 and 3 months after surgery, and the difference was statistically significant (*P* < 0.05). There was no statistically significant difference in VAS scores for low back pain between the two groups at 1 day and 6 months postoperatively (*P* > 0.05). See Table 3.

**Table 3:** Comparison of VAS scores for low back pain (independent samples *t*-test)

Group	Preoperative	One day after surgery	One month after surgery	Three month after surgery	June month after surgery
Open group	6.49 ± 1.50	3.81 ± 1.84	2.76 ± 1.12	1.76 ± 1.12	1.05 ± 0.74
Endoscopic group	6.75 ± 1.34	3.50 ± 0.98	2.19 ± 0.82	1.22 ± 0.71	0.75 ± 0.57
*t*	− 0.763	1.139	2.382	2.350	1.886
*P*	0.448	0.259	0.020	0.022	0.064

#### Leg pain VAS score

The difference in preoperative leg pain VAS scores between the two groups was not statistically significant (*P* > 0.05). Postoperative leg pain VAS scores were lower in both groups compared with preoperative scores, and the difference was statistically significant (*P* < 0.05). Leg pain VAS scores were lower in the UBE group than in the open group at 1, 3 and 6 months postoperatively, with a statistically significant difference (*P* < 0.05). The difference in leg pain VAS scores between the two groups at 1 day postoperatively was not statistically significant (*P* > 0.05). See Table 4.

**Table 4:** Comparison of VAS scores for leg pain (independent samples *t*-test)

Group	Preoperative	One day after surgery	One month after surgery	Three month after surgery	June month after surgery
Open group	6.46 ± 1.35	3.62 ± 1.26	2.22 ± 0.89	1.14 ± 0.63	0.78 ± 0.67
Endoscopic group	6.72 ± 1.25	3.31 ± 0.90	1.66 ± 0.65	0.72 ± 0.63	0.47 ± 0.62
*t*	− 0.825	1.160	2.948	2.728	2.010
*P*	0.412	0.250	0.004	0.008	0.048

#### ODI index

The difference in preoperative ODI indices between the two groups was not statistically significant (*P* > 0.05). The postoperative ODI index was lower in both groups compared to the preoperative period, and the difference was statistically significant (*P* < 0.05). The ODI index was lower in the UBE group than in the open PLIF group at 1 day and 1 month after surgery, with a statistically significant difference (*P* < 0.05). There was no statistically significant difference in the ODI index between the two groups at 3 and 6 months postoperatively (*P* > 0.05). See Table 5.

**Table 5:** Comparison of ODI indices (independent samples *t*-test)

Group	Preoperative	One day after surgery	One month after surgery	Three month after surgery	June month after surgery
Open group	43.12 ± 1.87	15.84 ± 1.67	11.08 ± 1.86	8.49 ± 1.52	6.16 ± 1.52
Endoscopic group	43.25 ± 1.88	14.56 ± 1.54	10.19 ± 1.55	7.88 ± 1.34	5.63 ± 1.39
*t*	− 0.254	1.160	2.144	1.760	1.562
*P*	0.800	0.250	0.036	0.083	0.132

#### Efficacy of modified MacNab

At 6 months postoperatively, according to the modified MacNab criteria, the excellent rate was 97.29% in the open PLIF group and 93.75% in the UBE group. The differences were not statistically significant (*P* > 0.05). See Table 6.

**Table 6:** Comparison of the efficacy of modified MacNab (Chi-square test)

Group	Excellent	good	average	poor	Excellent rate
Open group	23	13	1	0	97.29%
Endoscopic group	26	14	2	0	93.75%
X^2^			1.271		
*P*			0.530		

#### Situation of intervertebral fusion

Situation of intervertebral fusion was assessed using the Brantigan–Steffee criterion. The fusion of the operated lumbar segments was achieved in both groups with no statistically significant difference (*P* > 0.05). See Table 7.

**Table 7:** Comparison of intervertebral fusion (rank-sum test)

Group	A	B	C	D	E	Fusion rate
Open group	0	0	0	1	36	100%
Endoscopic group	0	0	0	2	30	100%
*z*			− 0.715			
*P*			0.474			

#### Radiological results

The differences in preoperative sagittal diameter of the spinal canal and preoperative intervertebral space height between the two groups were not statistically significant (*P* > 0.05).

The sagittal diameter of the spinal canal at 6 months postoperatively and the height of the intervertebral space at 6 months postoperatively increased in both groups compared with the preoperative period (*P* < 0.05). Comparison between groups, the differences in the sagittal diameter of the spinal canal at 6 months postoperatively and the intervertebral space height at 6 months postoperatively were not statistically significant (*P* > 0.05). The difference in postoperative adjacent vertebral instability between the two groups of patients was statistically significant (*P* < 0.05). See Tables 8 and 9.

**Table 8:** Comparison of adjacent vertebral instability (Chi-square test)

Group	Stable	Unstable
Open group	20	17
Endoscopic group	25	7
*x*^2^	4.383	
*P*	0.036

**Table 9:** Comparison of imaging findings (independent samples *t*-test)

Group	Sagittal diameter of the spinal canal		Intervertebral space height	
	Preoperative	June month after surgery	Preoperative	June month after surgery
Open group	9.84 ± 1.12	11.89 ± 0.81	6.08 ± 0.80	10.43 ± 1.04
Endoscopic group	9.91 ± 1.17	11.94 ± 0.88	6.13 ± 0.79	10.44 ± 1.06
*t*	− 0.248	− 0.225	− 0.299	− 0.200
*P*	0.805	0.823	0.820	0.984

### Postoperative complications

There was one case of incisional infection at 2 months postoperatively in the open PLIF group. In the UBE group, there was one case of incisional infection at 1 month postoperatively, one case of left lower extremity deep vein thrombosis at 20 days postoperatively and spastic numbness of both lower extremities at 1 month postoperatively. There was no statistically significant difference in postoperative complications between the two groups (*P* > 0.05). All improved after symptomatic treatment and bed rest. See Table 10.

**Table 10:** Comparison of postoperative complications (Chi-square test)

Group	Positive	Negative
Open group	1	36
Endoscopic group	2	30
*x*^2^	0.017	
*P*	0.898	

## Discussion

Lumbar spinal stenosis is a common degenerative disease in middle-aged and elderly people, and its clinical manifestations include lower limb numbness, pain, fatigue, intermittent claudication, recurrent lower back pain, with or without lower limb radiating pain, which seriously affects the quality of life of patients [11]. Usually non-surgical treatment is less effective, and disease progression to advanced stages often requires surgical intervention. In recent years, minimally invasive procedures in spine surgery have attracted attention in terms of reduced surgical burden and improved function. Interbody fusion is a commonly used surgical procedure in spinal surgery and was first described by Briggs and Milligan [12] in 1944 as posterior lumbar interbody fusion (PLIF). This procedure has now developed into a well-established surgical technique for the treatment of degenerative disk disease, spondylolisthesis and scoliosis [13, 14]. PLIF is performed by separating the muscle tissue from the spinal prominence and can also be performed by a percutaneous approach. This approach allows the surgeon to access the intravertebral canal to decompress the neural structures and visualize the entry point of the pedicle screw. Due to the large surgical field of view exposed during muscle contraction, on the one hand, it allows the operator to better visualize the nerve roots that need decompression, improving the success rate of the procedure; on the other hand, it tends to cause damage to the muscles, leading to postoperative pain and longer postoperative recovery time for patients [15, 16]. And with the widespread use of PLIF, it has also been exposed that it causes a lot of trauma, a lot of postoperative bleeding and a lot of postoperative complications. It also has an effect on lumbar spine stability and is prone to medically induced instability.

Related studies have shown that minimally invasive surgery is the best method to treat LSS [5]; with advances in surgical instrumentation and endoscopic techniques, minimally invasive spine surgery has rapidly evolved from small incisions to tubular or percutaneous endoscopy. The unilateral biportal endoscopy (UBE) is a percutaneous total endoscopic technique, and it is performed through two small separated surgical wounds on either side of the spinous process. Unlike other endoscopic methods, the UBE is not affected by the working channel, thus allowing for less restriction of surgical operations. Through continuous high-pressure saline flushing and high-definition arthroscopy, the surgeon can perform very precise decompression in a clear and magnified surgical view. It is a surgical procedure that combines open surgery with endoscopic spine surgery [17]. A biomechanical study showed that more than 50% of small joint injuries result in segmental instability [18], whereas the UBE technique preserves more than 80% of the proximal tuberosity and more than 90% of the contralateral tuberosity [19], and the protection of vertebral segmental stability is significantly effective. Arthroscopic discectomy was first improved in 1996 by De Antoni et al., they use separate instrument channels and place dual channels on the same side, allowing De Antoni et al. used independent instrument channels for the first time in 1996, placing the dual channels on the same side to allow unrestricted instrument activity. The technique has not made great strides, in large part because of the disadvantages of untimely removal of intraoperative haemorrhage, poor visualization, and susceptibility to postoperative spinal canal adhesions, in addition to the high demands placed on the surgeon by this procedure. In 2017, Korean scholar Eun proposed the use of arthroscopy in the observation channel to provide vision and insertion of minimally invasive discectomy in the operation channel based on the De Antoni procedure and successfully applied to the treatment of lumbar spondylolisthesis [21]. Endoscopic surgery is not without its drawbacks, the most critical issue is the learning curve, all minimally invasive techniques have high operative demands on the operator, and in contrast, the learning curve for the UBE technique for surgeons with no experience in endoscopic spine surgery is approximately 58 cases [22]. Therefore, surgeons need proper training and are more difficult to master than in open surgery.

Biomechanical changes in lumbar instability are associated with disk degeneration, which results in the loss of the physiological ability to absorb applied loading stresses and apply torsional resistance [9]. Numerous studies on lumbar intervertebral fusion adjacent to functional spinal units (FSUs) have reported: Increased postoperative stress between adjacent vertebral bodies can easily lead to poor stability of the FSU above or below the fused segment, include disk herniation, vertebral endplate degeneration, stenosis and hypertrophic small joint arthritis. The potential long-term complications of conventional spinal fusion are known as “adjacent segment degeneration (ASD)” [23], X-ray diagnosis is made by drawing a base line along the upper end plate of the vertebral body, two vertical lines from the upper posterior edge of the vertebral body and the lower posterior edge of the adjacent vertebral body, and the distance between the two vertical lines indicates the degree of slippage: If it exceeds 3 mm, it indicates a significant ASD [24]. The Iguchi study measured anteroposterior displacement and angulation between L4/5 on anterior flexion and posterior extension radiographs in 1090 patients with low back pain, and instability was diagnosed when the adjacent vertebrae were displaced by ≥ 3 mm and the intervertebral body angles were ≥ 10° [25]. Indirect signs of instability such as small joint asymmetry, unilateral saphenous stenosis, disk herniation and information related to predisposing anatomical factors can be observed by CT in the diagnosis of degenerative instability, and this leads to abnormal axial rotation of the lower vertebral body. These findings may be the ultimate cause of rotational sliding [26].

In this study, patients in both the open and endoscopic groups completed the procedure successfully. Intraoperative blood loss, time off the floor and time to hospital discharge were positively associated with the risk of postoperative infection in spine surgery. Back pain and leg pain scores, intraoperative bleeding and postoperative drainage were lower in the endoscopic group than in the open group. This shows that patients in the endoscopic group recovered more quickly than those in the open group. In addition, the incidence of postoperative adjacent vertebral instability was significantly lower in the endoscopic group than in the open group. The main reason may be that the endoscopic group has a better surgical field of view, which helps to preserve more of the posterior spinal column structures and reduces the damage to the paravertebral muscles and interference with the nerve roots by the surgical instruments.

The study showed that during a mean follow-up of 16 months, open and minimally invasive surgery for lumbar spinal stenosis results in significant changes in muscle area, and the development of back muscle dysfunction can disrupt normal function in other spinal areas and may lead to further development of spinal disease [27]. And the endoscopic approach has less impact on the muscle groups at the surgical site than the open approach, and these muscle groups are the paraspinatus, multifidus and erector spinae muscles [4]. Arja et al. showed that muscle function recovery in open surgery patients is incomplete at two months postoperatively, and it is therefore suspected that muscle damage from open surgery may jeopardize trunk muscle function and be involved in postoperative low back pain [28]. After the patient’s UBE procedure, significant enlargement of the cross-sectional area of the dural sac and the area of dural stenosis, early pain scores were significantly lower than in patients undergoing open surgery and lower incidence of associated paravertebral muscle injuries, and these suggest that the UBE procedure has good decompression effects and may be an alternative to conventional microsurgical decompression [29]. In addition, the more small articular surfaces are preserved, the lower the risk of instability after decompression. Compared to open laminectomy, the incidence of segmental instability after endoscopic decompression is significantly lower even in patients with mild slippage [30–32]. And, the overall complication rate of UBE surgery for lumbar spinal stenosis was 8.1% [33]; of these, dural tears are the most common complication, with an incidence of approximately 1.5–9.7% [34–37]. Although most dural tears are very small and can be managed conservatively, dural tears larger than 10 mm need to be repaired to prevent cerebrospinal fluid leakage and other sequelae [35]. Epidural hematoma is another common complication, one of the possible reasons for this is that the high intraoperative water pressure obscures the bleeding point, causing intraoperative visual field loss, and therefore, the intraoperative lavage hydraulic pressure should be optimally controlled at 25–30 mmHg [38]. In conclusion, UBE treatment for LSS can effectively improve the efficiency and shorten the operation time, and has the advantages of less trauma, less bleeding and significant improvement of postoperative pain symptoms, and a much lower incidence of lumbar instability [39, 40].

No dural tears occurred in either group of cases in this study. There was one case of incisional infection at 2 months postoperatively in the open PLIF group. In the UBE group, there were one case of incisional infection 1 month after surgery and one case of left lower limb deep vein thrombosis 20 days after surgery and spasticity and numbness of both lower limbs 1 month after surgery. All improved after symptomatic treatment and bed rest.

The advent of minimally invasive surgery has greatly accelerated the development of treatment for spine-related disorders. These new minimally invasive methods seek similar efficacy to traditional methods. The purpose of this study was to quantify the effect of open surgery and minimally invasive surgery on adjacent vertebral instability after treatment of lumbar spinal stenosis using radiological data. Although only one data set was used, these preliminary results provide some insight into the effect of both surgical approaches on postoperative adjacent vertebral instability. By comparing UBE technology with traditional open convergence technology, we found the former to be effective in the treatment of lumbar spinal stenosis, with the advantages of minimally invasive, adequate decompression of the spinal canal, and not affecting spinal stability. However, because of the limited sample size and short follow-up period, larger studies and longer follow-up are needed to confirm this finding.

## Data Availability

All the data in this study came from Changzhou First People’s Hospital, and all the data were recorded through the contemporaneous preservation of documents.
